# The Angular Momentum Dilemma and Born–Jordan Quantization

**DOI:** 10.1007/s10701-016-0041-8

**Published:** 2016-10-07

**Authors:** Maurice A. de Gosson

**Affiliations:** Faculty of Mathematics (NuHAG), University of Vienna, Wien, Austria

**Keywords:** Weyl quantization, Born–Jordan quantization, Angular momentum

## Abstract

The rigorous equivalence of the Schrödinger and Heisenberg pictures requires that one uses Born–Jordan quantization in place of Weyl quantization. We confirm this by showing that the much discussed “ angular momentum dilemma” disappears if one uses Born–Jordan quantization. We argue that the latter is the only physically correct quantization procedure. We also briefly discuss a possible redefinition of phase space quantum mechanics, where the usual Wigner distribution has to be replaced with a new quasi-distribution associated with Born–Jordan quantization, and which has proven to be successful in time-frequency analysis.

## Introduction

To address quantization problems in these “ Times of Entanglement” is not very fashionable: everything seems to have been said about this old topic, and there is more or less a consensus about the best way to quantize a physical system: it should be done using the Weyl transformation. The latter, in addition to being relatively simple, enjoys several nice properties, one of the most important being its “ symplectic covariance”, reflecting, at the quantum level, the canonical covariance of Hamiltonian dynamics. Things are, however, not that simple. If one insists in using Weyl quantization, one gets inconsistency, because the Schrödinger and Heisenberg pictures are then not equivalent. Dirac already notes in the Abstract to his paper [9] that “...*the Heisenberg picture is a good picture, the Schrödinger picture is a bad picture, and the two pictures are not equivalent*...”. This observation has also been confirmed by Kauffmann’s [17] interesting discussion of the non-physicality of Weyl quantization. This non-physicality is made strikingly explicit on an annoying contradiction known as the “ angular momentum dilemma”: the Weyl quantization of the squared classical angular-momentum is not the squared quantum angular momentum operator, but it contains an additional term $\frac{ 3}{2}\hbar ^{2}$. This extra term is actually physically significant, since it accounts for the nonvanishing angular momentum of the ground-state Bohr orbit in the hydrogen atom. This contradiction has been noted by several authors^1^, to begin with Linus Pauling in his *General Chemistry *[20]; it is also taken up by Shewell [21], and discussed in detail by Dahl and Springborg [7] and by Dahl and Schleich [8].

It turns out that we have shown in a recent work [13] that the Schrö dinger and Heisenberg pictures cannot be equivalent unless we use the quantization rule proposed by Born and Jordan [3, 4] in 1925, and which predates Weyl’s rule [22] by almost two years. We will see that using the BJ rule allows to eliminate the angular momentum, which seems to confirm that Weyl quantization should be replaced with Born–Jordan (BJ) quantization in the Schrödinger picture. Now, at first sight, this change of quantization rules does not lead to any earthshaking consequences for the Schrödinger picture, especially since one can prove [12] that BJ and Weyl quantizations coincide for all Hamiltonian functions of the type “ kinetic energy + potential” or, more generally, for Hamiltonian functions of the type

$$\begin{aligned} H(x,p,t)=\sum _{j=1}^{n}\frac{1}{2m_{j}}\left( p_{j}-A_{j}(x,t)\right) ^{2}+V(x,t) \end{aligned}$$

even when the potentials $A=(A_{1},\ldots ,A_{n})$ and *V* are irregular (we are using generalized coordinates $x=(x_{1},\ldots ,x_{n})$, $p=(p_{1},\ldots ,p_{n})$). One can therefore wonder whether it is really necessary to write yet another paper on quantization rules, just to deal with a quasi-philosophical problem (the equivalence of two pictures of quantum mechanics) and one little anomaly (the angular momentum dilemma, we will discuss below). The quantum world is however more subtle than that. The problem is that if we stick to Weyl quantization for general systems, another inconsistency appears, which could have far-reaching consequences. It is due to the fact the commonly used phase space picture of quantum mechanics, where the Wigner distribution plays a central role, is intimately related to Weyl quantization. In short, if we change the quantization rules, we also have to change the phase space picture, thus leading not only to a redefinition of the Wigner distribution, but also to substantial changes in related phase space objects, such as, for instance the Moyal product of two observables, which is at the heart of deformation quantization.

## BJ Versus Weyl: The Case of Monomials

Born, Jordan, and Heisenberg proved in [4] that the only way to quantize polynomials in a way consistent with Heisenberg’s ideas was to use the rule

1 $$\begin{aligned} p_{j}^{s}x_{j}^{r}\overset{\mathrm {BJ}}{\longrightarrow }\frac{1}{s+1} \sum _{\ell =0}^{s}\widehat{p}_{j}^{s-\ell }\widehat{x}_{j}^{r}\widehat{p} _{j}^{\ell }; \end{aligned}$$

or, equivalently,

2 $$\begin{aligned} p_{j}^{s}x_{j}^{r}\overset{\mathrm {BJ}}{\longrightarrow }\frac{1}{r+1} \sum _{j=0}^{r}\widehat{x}_{j}^{r-j}\widehat{p}_{j}^{s}\widehat{x}_{j}^{j} \end{aligned}$$

(see our paper [13] for a careful analysis of their argument). The BJ quantization is thus the *equally weighted* average of all the possible operator orderings. Weyl [22] proposed, independently, some time later a very general rule: elaborating on the Fourier inversion formula, he proposed that the quantization $\widehat{A}$ of a classical observable *a*(*x*, *p*) should be given by

3 $$\begin{aligned} \widehat{A}=\left( \tfrac{1}{2\pi \hbar }\right) ^{n}\iint Fa(x,p)e^{\frac{i}{ \hbar }(x\widehat{x}+p\widehat{p})}d^{n}xd^{n}p \end{aligned}$$

where *Fa*(*x*, *p*) is the Fourier transform of *a*(*x*, *p*) and $d^{n}x=dx_{1}\cdot \cdot \cdot dx_{n}$, $d^{n}p=dp_{1}\cdot \cdot \cdot dp_{n}$; applying this rule to monomials yields (McCoy [19])

4 $$\begin{aligned} p_{j}^{s}x_{j}^{r}\overset{\mathrm {Weyl}}{\longrightarrow }\frac{1}{2^{s}} \sum _{\ell =0}^{s}\left( {\begin{array}{c}s\\ \ell \end{array}}\right) \widehat{p}_{j}^{s-\ell }\widehat{x}_{j}^{r} \widehat{p}_{j}^{\ell }. \end{aligned}$$

It turns out that both the BJ and Weyl rules coincide as long as $s+r\le 2$, but they are different as soon as $s\ge 2$ and $r\ge 2$. For instance, to *xp* corresponds $\frac{1}{2}(\widehat{x}\widehat{p}+\widehat{p}\widehat{x}$ ) in both cases, but

$$\begin{aligned}&x^{2}p^{2}\overset{\mathrm {BJ}}{\longrightarrow }\frac{1}{3}(\widehat{x}^{2} \widehat{p}^{2}+\widehat{x}\widehat{p}^{2}\widehat{x}+\widehat{p}^{2} \widehat{x}^{2}) \\&x^{2}p^{2}\overset{\mathrm {Weyl}}{\longrightarrow }\frac{1}{4}(\widehat{x} ^{2}\widehat{p}^{2}+2\widehat{x}\widehat{p}^{2}\widehat{x}+\widehat{p}^{2} \widehat{x}^{2}) \end{aligned}$$

and both expressions differ by the quantity $\frac{1}{2}\hbar ^{2}$ as is easily checked by using several times the commutation rule $[\widehat{x}, \widehat{p}]=i\hbar$. We now make the following essential remark: let $\tau$ be a real number, and consider the somewhat exotic quantization rule

5 $$\begin{aligned} p_{j}^{s}x_{j}^{r}\overset{\tau }{\longrightarrow }\sum _{\ell =0}^{s}\left( {\begin{array}{c}s\\ \ell \end{array}}\right) (1-\tau )^{\ell }\tau ^{s-\ell }\widehat{p}_{j}^{s-\ell }\widehat{x}_{j}^{r} \widehat{p}_{j}^{\ell }. \end{aligned}$$

Clearly, the choice $\tau =\frac{1}{2}$ immediately yields Weyl’s rule (4); if we integrate the right-hand side of (5), then we get at once the BJ rule (2) since we have

$$\begin{aligned} \int _{0}^{1}(1-\tau )^{\ell }\tau ^{s-\ell }d\tau =\frac{(s-\ell )!\ell !}{(s+1)!} \end{aligned}$$

in view of the standard properties of the beta function and

$$\begin{aligned} \left( {\begin{array}{c}s\\ \ell \end{array}}\right) =\frac{s!}{\ell !(s-\ell )!}. \end{aligned}$$

This remark will allows us to define BJ quantization for arbitrary observables below.

## Generalized BJ Quantization

Our definition of BJ-quantization is inspired by earlier work in signal theory by Boggiato and his coworkers [1, 2]; for the necessary background in Weyl correspondence we refer to Littlejohn’s seminal paper [18], whose notation we use here (also see [10, 11]). Let $a=a(x,p)$ be an observable; writing $z=(x,p)$ the Weyl operator $\widehat{A}=\mathrm{Op}_{\mathrm {W}}(a)$ defined by formula (3) can also be written, performing the change of variables $(x,p)\longrightarrow (p,-x)$, as

6 $$\begin{aligned} \widehat{A}\psi =\left( \tfrac{1}{2\pi \hbar }\right) ^{n}\int a_{\sigma }(z) \widehat{T}(z)\psi d^{2n}z \end{aligned}$$

where $$d^{2n}z=d^{n}xd^{n}p$$ and

7 $$\begin{aligned} a_{\sigma }(z)=\left( \tfrac{1}{2\pi \hbar }\right) ^{n}\int e^{-\frac{i}{\hbar } \sigma (z,z^{\prime })}a(z^{\prime })d^{2n}z^{\prime } \end{aligned}$$

is the symplectic Fourier transform of *a* (see [10, 11, 18]). Here $\widehat{T}(z)=e^{-i\sigma (\widehat{z} ,z)/\hbar }$ is the usual Heisenberg–Weyl operator; $\sigma$ is the standard symplectic form defined by $\sigma (z,z^{\prime })=px^{\prime }-p^{\prime }x$ if $z=(x,p)$, $z^{\prime }=(x^{\prime },p^{\prime })$. The natural generalization of the $\tau$-rule (5) is obtained [12, 15] by replacing $\widehat{T}(z)$ with $=e^{-i\sigma _{\tau }( \widehat{z},z)/\hbar }$ where

$$\begin{aligned} \widehat{T}_{\tau }(z)=\exp \left( \frac{i}{2\hbar }(2\tau -1)px\right) \widehat{ T}(z); \end{aligned}$$

integrating for $0\le \tau \le 1$ one gets the Born–Jordan operator associated with the observable *a*:

8 $$\begin{aligned} \widehat{A}_{\mathrm {BJ}}\psi =\left( \tfrac{1}{2\pi \hbar }\right) ^{n}\int a_{\sigma }(z)\Theta (z)\widehat{T}(z)\psi d^{2n}z \end{aligned}$$

where

9 $$\begin{aligned} \Theta (z)=\int _{0}^{1}e^{\frac{i}{2\hbar }(2\tau -1)px}d\tau =\frac{ \sin (px/2\hbar )}{px/2\hbar } \end{aligned}$$

with $px=p_{1}x_{1}+\cdot \cdot \cdot +p_{n}x_{n}$. We thus have

10 $$\begin{aligned} (a_{\mathrm {W}})_{\sigma }(x,p)=a_{\sigma }(x,p)\frac{\sin (px/2\hbar )}{ px/2\hbar }. \end{aligned}$$

Taking the symplectic Fourier transform of $a_{\sigma }(z)\Theta (z)$, this means that $\widehat{A}_{\mathrm {BJ}}$ is the Weyl quantization, not of the observable *a*(*x*, *p*), but rather of the observable

11 $$\begin{aligned} a_{\mathrm {W}}=\left( \tfrac{1}{2\pi \hbar }\right) ^{n}a*\Theta _{\sigma }. \end{aligned}$$

The appearance of the function $\Theta$ in the formulas above is interesting; we have

$$\begin{aligned} \Theta (z)=\mathrm{sinc}(px/2\hbar ) \end{aligned}$$

where $\mathrm{sinc}$ is Whittaker’s *sinus cardinalis* function familiar from Fraunhofer diffraction [5].^2^

We now make an important remark: suppose that we split the phase space point (*x*, *p*) into two sets of independent coordinates $z^{\prime }=(x^{\prime },p^{\prime })$ and $z^{\prime \prime }=(x^{\prime \prime },p^{\prime \prime })$. Let $b(z^{\prime })$ be an observable in the first set, and $c(z^{\prime \prime })$ an observable in the second set, and define $a=b\otimes c$; it is an observable depending on the total set variables $(x,p)=(x^{\prime },x^{\prime \prime },p^{\prime },p^{\prime \prime })$. We obviously have $a_{\sigma }=b_{\sigma }\otimes c_{\sigma }$ and $\widehat{T} (z)=\widehat{T}(z^{\prime })\otimes \widehat{T}(z^{\prime \prime })$ (because the symplectic form $\sigma$ splits in the sum $\sigma ^{\prime }\oplus$ $\sigma ^{\prime \prime }$ of the two standard symplectic forms $\sigma ^{\prime }$ and $\sigma ^{\prime \prime }$ defined on, respectively, $z^{\prime }$ and $z^{\prime \prime }$ phase spaces). It follows from formula (6) that we have $\widehat{A}=\widehat{B}\otimes \widehat{C}$; *i.e.* Weyl quantization preserves the separation of two observables. This property is generically not true for Born–Jordan quantization: because of the presence in formula (8) of the function $\Theta (z)$ we cannot write the integrand as a tensor product, and hence we have in general

12 $$\begin{aligned} \widehat{A}_{\mathrm {BJ}}\ne \widehat{B}_{\mathrm {BJ}}\otimes \widehat{C}_{ \mathrm {BJ}} \end{aligned}$$

In this sense, Born–Jordan quantization “ entangles” quantum observables; it would certainly be interesting to study this phenomenon and its implications.

## The Angular Momentum Dilemma

Dahl and Springborg’s [7] argument we alluded to in the introduction boils down to the following observation for the electron in the hydrogen atom in its 1*s* state. Let

13 $$\begin{aligned} \widehat{\ell }=\left( \widehat{x}_{2}\widehat{p}_{3}-\widehat{x}_{3}\widehat{p} _{2}\right) \mathbf {i}+\left( \widehat{x}_{3}\widehat{p}_{1}-\widehat{x}_{1}\widehat{p} _{3}\right) \mathbf {j}+\left( \widehat{x}_{1}\widehat{p}_{2}-\widehat{x}_{2}\widehat{p} _{1}\right) \mathbf {k} \end{aligned}$$

be the angular momentum operator and

14 $$\begin{aligned} \widehat{\ell }^{2}=\big (\widehat{x}_{2}\widehat{p}_{3}-\widehat{x}_{3}\widehat{p }_{2}\big )^{2}+\big (\widehat{x}_{3}\widehat{p}_{1}-\widehat{x}_{1}\widehat{p} _{3}\big )^{2}+\big (\widehat{x}_{1}\widehat{p}_{2}-\widehat{x}_{2}\widehat{p}_{1}\big )^{2} \end{aligned}$$

its square. According to the Bohr model, the square $\ell ^{2}$ of the classical angular momentum

15 $$\begin{aligned} \ell =\big (x_{2}p_{3}-x_{3}p_{2},x_{3}p_{1}-x_{1}p_{3},x_{1}p_{2}-x_{2}p_{1}\big ) \end{aligned}$$

should have the value $\hbar ^{2}$, while it is zero in the Schrödinger picture. Thus, Dahl and Springborg contend, the “ dequantization” of $\widehat{\ell }^{2}$ should yield the Bohr value^3^. Now, “ dequantizing” $\widehat{\ell }^{2}$ using the Weyl transformation leads to the function $\ell ^{2}+\tfrac{3}{2}\hbar ^{2}$ (as was already remarked by Shewell [21], formula (4.10)), which gives the “ wrong” value $\tfrac{3}{2}\hbar ^{2}$ for the Bohr angular momentum. However, if we view $\widehat{\ell }^{2}$ as the Born–Jordan quantization of $\ell ^{2}$, then we recover the Bohr value $\hbar ^{2}$. Let us show this in some detail. It suffices of course to study one of the three terms appearing in the square of the vector (15 ), say

16 $$\begin{aligned} \ell _{3}^{2}=x_{1}^{2}p_{2}^{2}+x_{2}^{2}p_{1}^{2}-2x_{1}p_{1}x_{2}p_{2}. \end{aligned}$$

The two first terms in (16) immediately yield the operators $\widehat{ x}_{1}^{2}\widehat{p}_{2}^{2}$ and $\widehat{x}_{2}^{2}\widehat{p}_{1}^{2}$ (as they would in any realistic physical quantization scheme), so let us focus on the third term $a_{12}(z)=2x_{1}p_{1}x_{2}p_{2}$ (we are writing here $z=(x_{1},x_{2},p_{1},p_{2})$). Using the standard formula giving the Fourier transform of a monomial we get

17 $$\begin{aligned} a_{12,\sigma }(z)=2\hbar ^{4}(2\pi \hbar )^{2}\delta ^{\prime }(z) \end{aligned}$$

where we are using the notation

$$\begin{aligned} \delta (z)&\equiv \delta (x_{1})\otimes \delta (x_{2})\otimes \delta (p_{1})\otimes \delta (p_{2}), \\ \delta ^{\prime }(z)&\equiv \delta ^{\prime }(x_{1})\otimes \delta ^{\prime }(x_{2})\otimes \delta ^{\prime }(p_{1})\otimes \delta ^{\prime }(p_{2}). \end{aligned}$$

Expanding the function $\sin (px/2\hbar )$ in a Taylor series, we get

$$\begin{aligned} \Theta (z)=1+\sum _{k=1}^{\infty }\frac{(-1)^{k}}{(2k+1)!}\left( \frac{px}{ 2\hbar }\right) ^{2k} \end{aligned}$$

and hence, observing that $(px)^{2k}\delta ^{\prime }(z)=0$ for $k>1$,

18 $$\begin{aligned} a_{12,\sigma }(z)\Theta (z)=a_{12,\sigma }(z)\left( 1-\frac{(px)^{2}}{ 24\hbar ^{2}}\right) . \end{aligned}$$

Comparing the expressions (6) and (8), defining respectively the Weyl and Born–Jordan quantizations of *a*, it follows that the difference

$$\begin{aligned} \Delta (a_{12})=\mathrm{Op}_{\mathrm {BJ}}(a_{12})-\mathrm{Op} _{\mathrm {W}}(a_{12}) \end{aligned}$$

is given by

$$\begin{aligned} \Delta (a_{12})\psi&=\frac{1}{24\hbar ^{2}}\left( \frac{1}{2\pi \hbar } \right) ^{2}\int a_{12,\sigma }(z)(px)^{2}\widehat{T}(z)\psi d^{4}z \\&=\frac{\hbar ^{2}}{12}\int \delta ^{\prime }(z)(px)^{2}\widehat{T}(z)\psi d^{4}z. \end{aligned}$$

Using the elementary properties of the Dirac function we have

19 $$\begin{aligned} \delta ^{\prime }(z)(px)^{2}=2\delta (z) \end{aligned}$$

and hence

$$\begin{aligned} \Delta (a_{12})\psi =\frac{\hbar ^{2}}{6}\int \delta (z)\widehat{T}(z)\psi d^{4}z= \frac{\hbar ^{2}}{6}\psi \end{aligned}$$

the second equality because

$$\begin{aligned} \delta (z)\widehat{T}(z)=\delta (z)e^{-\frac{i}{\hbar }\sigma (\widehat{z} ,z)}=\delta (z). \end{aligned}$$

A similar calculation for the quantities $\Delta (a_{23})$ and $\Delta (a_{13})$ corresponding to the terms $\ell _{1}^{2}$ and $\ell _{2}^{2}$ leads to the formula

20 $$\begin{aligned} \mathrm{Op}_{\mathrm {BJ}}(\ell ^{2})-\mathrm{Op}_{\mathrm { W}}(\ell ^{2})=\tfrac{1}{2}\hbar ^{2}, \end{aligned}$$

hence, taking (14) into account:

21 $$\begin{aligned} \mathrm{Op}_{\mathrm {BJ}}(\ell ^{2})=\widehat{\ell }^{2}+\hbar ^{2} \end{aligned}$$

which is the expected result. We note that Dahl and Springborg [7] get the same operator $\widehat{\ell }^{2}+\hbar ^{2}$ by averaging the Weyl operator $\mathrm{Op}_{\mathrm {W}}(\ell ^{2})$ over what they call a “ classical subspace”; funnily enough the $\mathrm{sinc}$ function also appears in their calculations ([7], formula (40)). It would be interesting to see whether this is a mere coincidence, or if a hidden relation with BJ quantization already is involved in these calculations.

## The BJ-Wigner Transform

As we mentioned in the introduction, the phase space picture of a quantum system very much depends on the quantization procedure that is used. In the Wigner formalism, if $\widehat{A}_{\mathrm {W}}=\mathrm{Op}_{\mathrm {W}}(a)$,

22 $$\begin{aligned} \big \langle \psi |\widehat{A}_{\mathrm {W}}|\psi \big \rangle =\int a(z)W\psi (z)d^{2n}z \end{aligned}$$

where $\psi$ is normalized, and

$$\begin{aligned} W\psi (z)=\left( \tfrac{1}{2\pi \hbar }\right) ^{n}\int e^{-\frac{i}{\hbar } py}\psi \big (x+\tfrac{1}{2}y\big )\psi ^{*}\big (x-\tfrac{1}{2}y\big )d^{n}y \end{aligned}$$

is the usual Wigner quasi distribution [10, 11, 18]. As we have shown in [12], if we replace Weyl quantization of the classical observable *a* with its Born–Jordan quantization $\widehat{A}_{ \mathrm {BJ}}=\mathrm{Op}_{\mathrm {BJ}}(a)$, then formula (22) becomes

23 $$\begin{aligned} \big \langle \psi |\widehat{A}_{\mathrm {BJ}}|\psi \big \rangle =\int a(z)W_{\mathrm {BJ} }\psi (z)d^{2n}z \end{aligned}$$

where $W_{\mathrm {BJ}}\psi$ is given by the convolution formula

24 $$\begin{aligned} W_{\mathrm {BJ}}\psi (z)=\left( \tfrac{1}{2\pi \hbar }\right) ^{n}W\psi *\Theta _{\sigma } \end{aligned}$$

(this can easily be proven using formula (11)). Let us compare the expressions $\langle \psi |\widehat{A}_{\mathrm {W}}|\psi \rangle$ and $\langle \psi |\widehat{A}_{\mathrm {BJ}}|\psi \rangle$ where $\widehat{A}_{ \mathrm {W}}=\mathrm{Op}_{\mathrm {W}}(a)$ and $\widehat{A}_{\mathrm {BJ}}= \mathrm{Op}_{\mathrm {BJ}}(a)$. In view of Parseval’s theorem we can rewrite formulas (22) and (23) as

25 $$\begin{aligned} \big \langle \psi |\widehat{A}_{\mathrm {W}}|\psi \big \rangle&=\int a_{\sigma }(z)F_{\sigma }W\psi (z)d^{2n}z \end{aligned}$$

26 $$\begin{aligned} \big \langle \psi |\widehat{A}_{\mathrm {BJ}}|\psi \big \rangle&=\int a_{\sigma }(z)F_{\sigma }W_{\mathrm {BJ}}\psi (z)d^{2n}z. \end{aligned}$$

Since $F_{\sigma }W_{\mathrm {BJ}}\psi (z)=F_{\sigma }W\psi (z)\Theta (z)$ we have

$$\begin{aligned} \big \langle \psi |\widehat{A}_{\mathrm {W}}|\psi \big \rangle -\big \langle \psi |\widehat{A}_{ \mathrm {BJ}}|\psi \big \rangle = \int a_{\sigma }(z)F_{\sigma }W\psi (z)(1-\Theta (z))d^{2n}z. \end{aligned}$$

Let us apply this formula to the square $\ell ^{2}$ of the angular momentum. As above, we only have to care about the cross term (17); in view of formula (18) above we have

$$\begin{aligned} a_{\sigma }(z)(1-\Theta (z))=\frac{\hbar ^{2}}{6}(2\pi \hbar )^{2}\delta (z) \end{aligned}$$

and hence

$$\begin{aligned} \big \langle \psi |\widehat{A}_{\mathrm {W}}|\psi \big \rangle -\big \langle \psi |\widehat{A}_{ \mathrm {BJ}}|\psi \big \rangle = \frac{\hbar ^{2}}{6}(2\pi \hbar )^{2}\int \delta (z)F_{\sigma }W\psi (0)dz. \end{aligned}$$

Observing that

$$\begin{aligned} F_{\sigma }W\psi (0)=\left( \tfrac{1}{2\pi \hbar }\right) ^{2}\int W\psi (z)dz=\left( \tfrac{1}{2\pi \hbar }\right) ^{2} \end{aligned}$$

we finally get

$$\begin{aligned} \big \langle \psi |\widehat{A}_{\mathrm {BJ}}|\psi \big \rangle -\big \langle \psi |\widehat{A}_{ \mathrm {W}}|\psi \big \rangle =-\tfrac{1}{6}\hbar ^{2}. \end{aligned}$$

it follows, taking the two other cross-components of $\ell ^{2}$ into account, that we have

$$\begin{aligned} \big \langle \psi |\widehat{\ell }_{\mathrm {BJ}}^{2}|\psi \big \rangle -\big \langle \psi | \widehat{\ell }_{\mathrm {W}}^{2}|\psi \big \rangle =-\tfrac{1}{2}\hbar ^{2} \end{aligned}$$

and hence

27 $$\begin{aligned} \big \langle \psi |\widehat{\ell }_{\mathrm {BJ}}^{2}|\psi \big \rangle =\hbar ^{2} \end{aligned}$$

as predicted by Bohr’s theory.

## Discussion

We have tried to make it clear that to avoid inconsistencies one has to use BJ quantization instead of the Weyl correspondence in the Schrödinger picture of quantum mechanics. With hindsight, it somewhat ironic that the “ true” quantization should be the one which was historically the first to be proposed. There are, however, unexpected difficulties that appear; the mathematics of BJ quantization is not fully understood. For instance, the generalized Born–Jordan rule (8) does not implement a true “ correspondence”, as the Weyl rule does. In fact, it results from a deep mathematical theorem, Schwartz’s kernel theorem [11], that every quantum observable *A* which is sufficiently smooth can be viewed as the Weyl transform of some classical observable *a*, and conversely. However, this is not true of the BJ quantization scheme: it is not true that to every quantum observable (or “ operator”) one can associate a classical observable. In fact, rewriting formula (11) as

28 $$\begin{aligned} (a_{\mathrm {W}})_{\sigma }(x,p)=a_{\sigma }(x,p)\frac{\sin (px/2\hbar )}{ px/2\hbar } \end{aligned}$$

we see that we cannot, in general, calculate $a_{\sigma }(x,p)$ (and hence *a*(*x*, *p*)) if we know the Weyl transform $a_{\mathrm {W}}$ of *A*, and this because the function $\Theta (x,p)=\sin (px/2\hbar )/(px/2\hbar )$ has infinitely many zeroes: $\Theta (x,p)=0$ for all phase space points (*x*, *p*) such that $p_{1}x_{1}+\cdot \cdot \cdot +p_{n}x_{n}=0$. We are thus confronted with a difficult division problem; see [6]. It is also important to note that we loose uniqueness of quantization when we use the Born–Jordan “ correspondence”: if $(a_{ \mathrm {W}})_{\sigma }(x,p)=0$ there are infinitely many Weyl operators who verify (28). These issues, which might lead to interesting developments in quantum mechanics, are discussed in detail in our book [14]. The BJ-Wigner transform and its relation with what we call “ Born–Jordan quantization” has been discovered independently by Boggiatto and his coworkers [1, 2] who were working on certain questions in signal theory and time-frequency analysis; they show—among other things—that the spectrograms obtained by replacing the standard Wigner distribution by its modified version $W_{\mathrm {BJ}}\psi$ are much more accurate. The properties of $W_{\mathrm {BJ}}\psi$ are very similar to those of $W\psi$; it is always a real function, and it has the “ right” marginals and can thus be treated as a quasi-distribution, exactly as the traditional Wigner distribution does.

## Footnotes

1 It is also mentioned in Wikipedia’s article [16] on geometric quantization.

2 I thank Basil Hiley for having drawn my attention to this fact.

3 Of course, their argument is heuristic, because there is *in general* no relation between the eigenvalues of a quantum operator and the values of the corresponding classical observable.
